# FAM83 family oncogenes are broadly involved in human cancers: an integrative multi‐omics approach

**DOI:** 10.1002/1878-0261.12016

**Published:** 2017-01-09

**Authors:** Antoine M. Snijders, Sun‐Young Lee, Bo Hang, Wenshan Hao, Mina J. Bissell, Jian‐Hua Mao

**Affiliations:** ^1^ Biological Systems and Engineering Division Lawrence Berkeley National Laboratory CA USA; ^2^ Nanjing Biotech and Pharmaceutical Valley Development Center China

**Keywords:** copy number variation, FAM83 family, gene mutation, multi‐omics approach, oncogenes

## Abstract

The development of novel targeted therapies for cancer treatment requires identification of reliable targets. FAM83 (‘family with sequence similarity 83’) family members A, B, and D were shown recently to have oncogenic potential. However, the overall oncogenic abilities of FAM83 family genes remain largely unknown. Here, we used a systematic and integrative genomics approach to investigate oncogenic properties of the entire FAM83 family members. We assessed transcriptional expression patterns of eight FAM83 family genes (FAM83A‐H) across tumor types, the relationship between their expression and changes in DNA copy number, and the association with patient survival. By comparing the gene expression levels of FAM83 family members in cancers from 17 different tumor types with those in their corresponding normal tissues, we identified consistent upregulation of FAM83D and FAM83H across the majority of tumor types, which is largely driven by increased DNA copy number. Importantly, we found also that a higher expression level of a signature of FAM83 family members was associated with poor prognosis in a number of human cancers. In breast cancer, we found that alterations in FAM83 family genes correlated significantly with TP53 mutation, whereas significant, but inverse correlation was observed with PIK3CA and CDH1 (E‐cadherin) mutations. We also identified that expression levels of 55 proteins were significantly associated with alterations in FAM83 family genes including a decrease in GATA3, ESR1, and PGR proteins in tumors with alterations in FAM83. Our results provide strong evidence for a critical role of FAM83 family genes in tumor development, with possible relevance for therapeutic target development.

**Abbreviations**
DNA: deoxyribonucleic acid
FAM83: family with sequence similarity 83
GEO: gene expression omnibus
GTEx: Genotype‐Tissue Expression
HPM: Human Proteome Map
HPA: Human Protein Atlas
mRNA: messenger ribonucleic acid
NCBI: National Center for Biotechnology Information
RPKM: reads per kilobase of transcript per million mapped reads
SCC: squamous cell carcinoma
shRNA: short hairpin RNA
TCGA: The Cancer Genome Atlas
TKI: tyrosine kinase inhibitor

## 1 Introduction

Recent studies have demonstrated that some of the ‘family with sequence similarity 83’ (FAM83) family members have oncogenic properties and have significantly elevated expression levels in multiple human tumor types, including breast cancers (Lee *et al*., 2012) (Cipriano *et al*., 2012) (Wang *et al*., 2013b). The eight FAM83 family members are characterized by a highly conserved domain of unknown function (Cipriano *et al*., 2014b). FAM83A is downstream of EGFR and PI3K pathways and is associated with RAS/RAF/MEK/ERK and PI3K/AKT/mTOR pathways (Lee *et al*., 2012). Importantly, FAM83A interacts with c‐RAF and PI3K p85 components of the EGFR pathway, and FAM83A expression was correlated with tumor growth rate (Lee *et al*., 2012). It was shown also that when tumor cells with high levels of EGFR were injected into mice and treated with EGFR inhibitors, gene expression and DNA copy number of endogenous FAM83A increase, making surviving tumor cells resistant to therapy (Lee *et al*., 2012). FAM83B has been implicated as a mediator of EGFR‐RAS‐MAPK‐driven oncogenic transformation, and an increase in *FAM83B* gene expression was also observed in different tumor types, associated with increased tumor grade and decreased patient survival (Cipriano *et al*., 2012). *FAM83D* was also amplified and overexpressed in many types of human cancer, and its expression significantly correlated with patient outcome (Walian *et al*., 2016). Forced expression of FAM83D in nonmalignant cells in culture promoted proliferation and invasion of breast cancer cells and downregulated the expression of tumor suppressor gene, FBXW7 (Wang *et al*., 2013b). An insertional mutagenesis screen in an orthotopic mouse model identified FAM83H as one of eleven genes that promote androgen‐independent prostate cancer (Nalla *et al*., 2016). It has also been shown recently that FAM83H regulates the organization of the keratin cytoskeleton and formation of desmosomes (Kuga *et al*., 2013, 2016). However, the detailed mechanism of action of FAM83 family members in human cancer still remains to be discovered.

The availability of large cancer genomic data sets allows for unbiased approaches to assess oncogenic properties of genes. Gene transcript‐based signatures that predict prognosis have successfully been developed for many different tumor types. Here, we combined available cancer databases to identify oncogenic properties of FAM83 family genes across tumor types by combining gene transcript, DNA copy number, and mutation status with their ability to predict prognosis. Our strategy provides further evidence for a critical role of FAM83 family genes in tumor development, which could be exploited to design better treatment strategies.

## 2 Methods

Gene transcript data (RPKM: RNA sequencing reads per kilobase of transcript per million mapped reads) of FAM83 family genes across normal tissue types were obtained from the Genotype‐Tissue Expression (GTEx) project (Carithers *et al*., 2015; Mele *et al*., 2015). Gene expression data obtained from GTEx were compared to human proteome data obtained from the Human Proteome Map (HPM; http://www.humanproteomemap.org) and the Human Protein Atlas (HPA; http://www.proteinatlas.org) (Kim *et al*., 2014; Uhlen *et al*., 2015). Gene transcript data of normal and tumor tissues across seventeen different tumor types were obtained from the National Center for Biotechnology Information (NCBI) Gene Expression Omnibus (GEO) (brain: GSE4290; breast: GSE10780, GSE21422, GSE29044, and GSE3744; colon: GSE8671; gastric: GSE13911; head‐and‐neck: GSE6791; liver: GSE6764, GSE40367, GSE45267, GSE55092, and GSE62232; nasopharyngeal: GSE12452; lung: GSE31210, GSE19804, and GSE19188; ovarian: GSE14407; cervical: GSE6791; pancreatic: GSE16515; and prostate cancer: GSE3325). Fold change was calculated for each gene, and significance was tested using t‐test (*P *< 0.05). Genomic alterations and mRNA expression levels for TCGA studies were obtained from cBioPortal (Cerami *et al*., 2012; Gao *et al*., 2013). We used a rank‐based nonparametric test (Kruskal–Wallis) to determine whether gene expression levels were significantly different between copy number groups (*P *< 0.01 was used as a threshold for significance). All information concerning these samples can be downloaded from cBioPortal (http://www.cbioportal.org/data_sets.jsp). Survival multivariate analysis and risk assessment for individual FAM83 family genes and gene signatures in human cancer data sets were performed using SurvExpress (Aguirre‐Gamboa *et al*., 2013) in the following data sets: ovarian (GSE9891 and GSE32062), head‐and‐neck (TCGA and E‐MTAB‐1328), breast (TCGA and GSE20685), liver (TCGA), lung adenocarcinoma (TCGA), lung squamous cell carcinoma (TCGA), pancreatic (GSE28735 and GSE21501), stomach (TCGA), colon (TCGA and GSE17536), brain low‐grade glioma (TCGA), and brain glioblastoma multiforme (GSE16011). Mutation and protein‐level enrichment data were downloaded from cBioPortal. Gene ontology enrichment analysis was performed using the Web‐based gene set analysis toolkit (BH‐corrected *P *< 0.05 was used as a threshold for significance) (Wang *et al*., 2013a; Zhang *et al*., 2005).

## 3 Results

### 3.1 Expression levels of FAM83 family genes vary widely across human *normal* tissues

**Figure 1:**
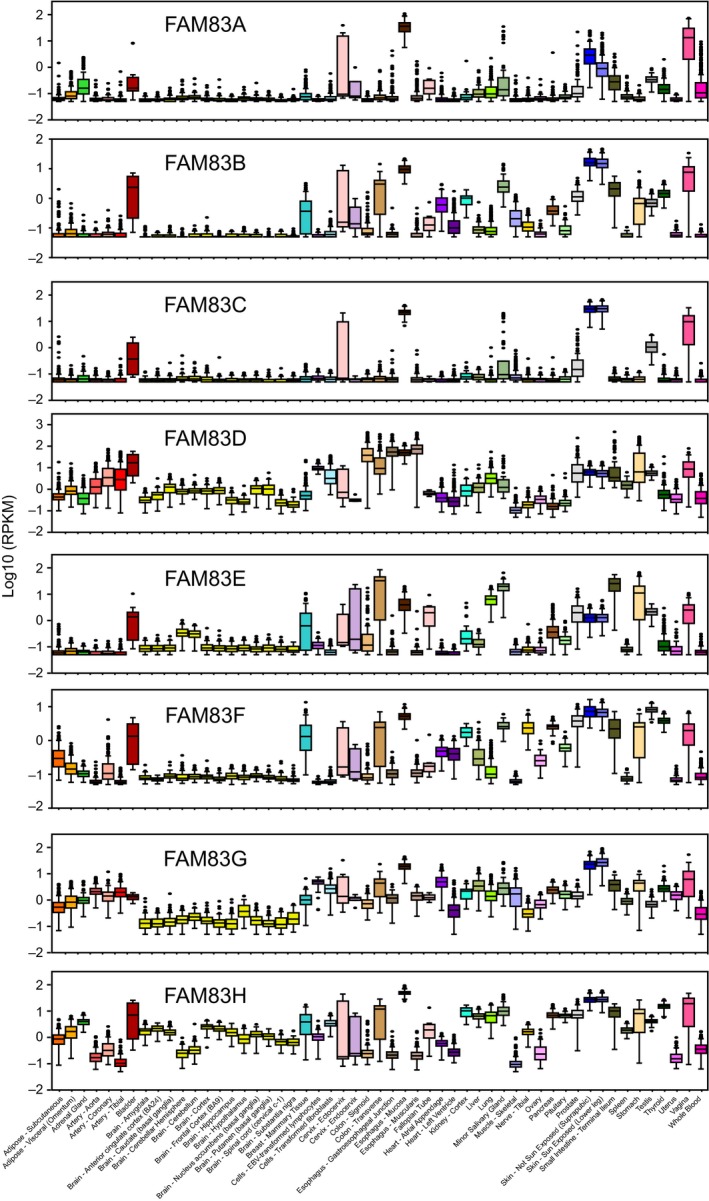
Gene expression levels of FAM83 family genes vary across normal human tissue types. Box plots of RPKM levels of FAM83 family genes across 53 different tissue types.

To gain insight into the oncogenic role of FAM83 family members in human cancer, we conducted a meta‐analysis of their gene expression in human normal and cancer tissues using publically available and The Cancer Genome Atlas (TCGA) data (Fig. S1). From 53 human *normal* tissue types available from the Genotype‐Tissue Expression (GTEx) database (Mele *et al*., 2015), we observed a wide range of expression levels in different tissues (Fig. 1). It is found that all eight FAM83 family members transcriptionally expressed at relatively high level in the normal mucosa of the esophagus, bladder, cervix, vagina, and skin while at relatively low levels in brain (Fig. 1). In contrast, specific gene expression patterns of FAM83 family member were observed in normal breast tissue (Fig. 1). In addition, we obtained protein‐level data of FAM83 family members across normal human tissues from the Human Proteome Map (HPM; http://www.humanproteomemap.org) and the Human Protein Atlas (HPA; http://www.proteinatlas.org) and observed general agreement between gene transcript and protein levels (Fig. S2).

### 3.2 FAM83D and FAM83H are consistently upregulated across human tumor types

**Figure 2:**
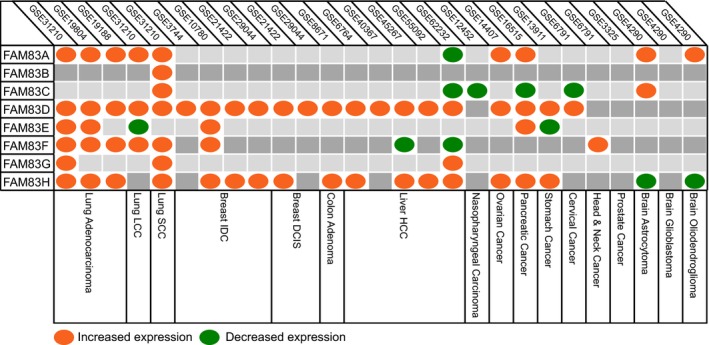
FAM83D and FAM83H are consistently overexpressed across human tumor types. Gene expression of FAM83 family genes across different tumor types. Increased or decreased gene expression (1.5‐fold; adjusted *P *< 0.05) is shown in orange and green, respectively.

We collected gene transcript data of normal and tumor tissues across 17 different tumor types represented by 27 independent data sets (Fig. 2; Table S1). The significant differential expression of all eight FAM83 family genes (Table S1) was assessed by a fold‐change cutoff of 1.5 and adjusted *P*‐value* *< 0.05 (Fig. 2). Transcriptional levels of FAM83 members were frequently elevated in tumors, consistent with their proposed oncogenic properties. Specifically, FAM83D and FAM83H were upregulated in 21 of 27 data sets (78%) and 16 of 27 data sets (59%) data sets, respectively. On the other hand, FAM83B, C, E, and G were upregulated in less than five tissue types each (Fig. 2).

### 3.3 DNA copy number increase is a potential mechanism for increased expression of FAM83D and H

**Figure 3:**
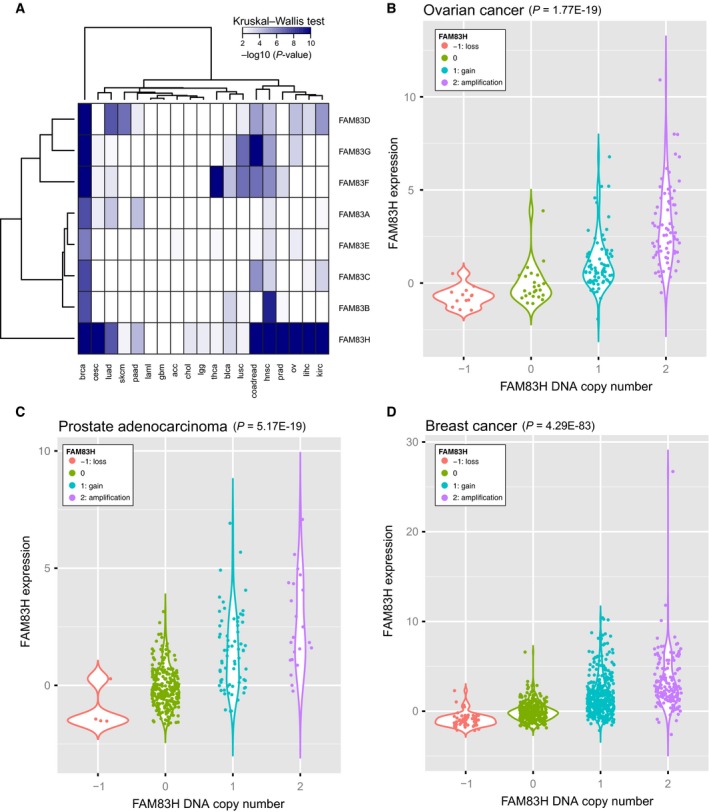
DNA copy number of FAM83D and FAM83H is strongly correlated with gene expression. (A) Heatmap of the *P*‐values of the relationship between DNA copy number and gene expression (Kruskal–Wallis test; *P *< 0.01). Tumor type abbreviations: breast: breast invasive carcinoma; cesc: cervical squamous cell carcinoma and endocervical adenocarcinoma; ucec: uterine corpus endometrial carcinoma; skcm: skin cutaneous melanoma; coadread: colorectal adenocarcinoma; stad: stomach adenocarcinoma; luad: lung adenocarcinoma; hnsc: head‐and‐neck squamous cell carcinoma; kirc: kidney renal clear cell carcinoma; blca: urothelial bladder carcinoma; paad: pancreatic adenocarcinoma; prad: prostate adenocarcinoma; lihc: liver hepatocellular carcinoma; ov: ovarian serous cystadenocarcinoma; lusc: lung squamous cell carcinoma; lgg: low‐grade glioma; gbm: glioblastoma multiforme; kirp: kidney renal papillary cell carcinoma; pcpg: pheochromocytoma and paraganglioma; laml: acute myeloid leukemia; thca: thyroid carcinoma. (B–D) Box plot of the relationship between DNA copy number and gene expression for FAM83H in ovarian cancer (panel B; *P *= 1.77E‐19), prostate adenocarcinoma (panel B; *P *= 5.17E‐19), and breast cancer (panel C; *P *= 4.29E‐83). The kernel probability density of the data at different values is shown.

The Cancer Genome Atlas (TCGA) data were analyzed to search for the possible mechanism by which FAM83 family genes are upregulated in cancer. Whereas mutations in FAM83 family genes were relatively rare, DNA copy number alterations encompassing FAM83 genes were frequently observed in many human tumor types (Table 1). Changes in DNA copy number are often observed in tumors, and DNA copy number aberrations are one of the mechanisms that can result in a change in gene expression in tumor progression. It was shown previously that high‐level gene amplifications are under continuous selection pressures, and when selection pressure is removed, amplifications are not maintained and eventually disappear (Meinkoth *et al*., 1987; Snijders *et al*., 2008). Thus, it is possible that DNA copy number amplification is focused on those genes that are important for tumor development. For each of the eight FAM83 family genes, we used a rank‐based nonparametric test to determine whether the transcriptional expression levels are significantly associated with their copy number. A heatmap of the *P*‐values of the rank‐based test is shown in Fig. 3A for all eight family members across 19 tumor types, and we observed a strong association between DNA copy number and gene expression for all FAM83 family genes in breast and head‐and‐neck cancer (*P *< 0.01; Fig. 3A). In addition, FAM83D and FAM83H showed a significant association between DNA copy number and expression in 10/19 and 16/19 tumor types, respectively (*P *< 0.01). Examples of the association of DNA copy number and gene expression are shown for FAM83H in ovarian cancer (*P *= 1.77E‐19), prostate adenocarcinoma (*P *= 5.17E‐19), and breast cancer (*P *= 4.29E‐83) (Fig. 3B–D). Thus, genomic DNA copy number increase in FAM83D and H is expected to contribute to tumor evolution by copy number‐induced alterations in gene expression.

**Table 1:** Summary of DNA copy number increase (gain) and decrease (loss) and mutation for FAM83 family genes across 19 tumor types

Tumor type	Total number of tumors	FAM83A			FAM83B			FAM83C			FAM83D			FAM83E			FAM83F			FAM83G			FAM83H		
		Gain	Loss	Mutation	Gain	Loss	Mutation	Gain	Loss	Mutation	Gain	Loss	Mutation	Gain	Loss	Mutation	Gain	Loss	Mutation	Gain	Loss	Mutation	Gain	Loss	Mutation
acc	75	34	10	0	14	12	1	43	2	1	42	2	0	44	5	1	2	39	0	3	23	0	33	11	5
blca	126	71	7	0	30	27	2	86	1	3	86	2	2	53	15	2	23	52	1	31	49	4	68	8	6
brca	960	593	31	3	221	148	5	419	49	2	404	56	0	206	177	2	95	436	3	100	495	3	555	61	2
cesc	190	68	15	0	36	29	3	83	2	1	77	5	1	52	20	1	27	44	0	15	52	3	70	15	1
chol	35	15	1	0	8	5	0	13	2	0	13	2	0	6	5	0	6	5	0	5	9	0	15	1	0
coadread	195	99	4	3	28	8	4	144	0	5	144	0	4	33	6	0	2	48	0	2	96	2	96	7	1
gbm	136	18	12	0	6	25	3	51	3	0	51	3	1	32	25	0	10	58	0	14	16	0	20	11	2
hnsc	279	203	2	0	55	38	4	124	12	4	114	17	0	40	54	1	66	41	0	41	53	5	200	4	5
kirc	413	65	40	2	11	71	6	99	0	2	99	0	1	42	4	0	31	34	0	18	30	3	66	40	1
laml	163	20	0	0	0	0	0	1	3	0	0	3	0	5	3	0	9	2	0	0	14	0	20	0	0
lgg	283	57	6	1	8	18	4	26	1	0	26	1	0	13	137	0	10	36	0	9	16	1	53	17	1
lihc	190	110	10	0	70	7	2	62	7	2	63	6	1	47	24	2	32	53	0	30	75	0	108	10	4
luad	230	139	13	3	79	32	11	100	23	5	89	32	4	28	82	0	31	97	1	40	94	3	129	19	6
lusc	178	124	6	1	57	28	8	96	13	4	93	18	5	65	43	2	85	34	0	22	103	1	121	3	2
ov	182	140	10	0	55	49	0	103	3	0	95	14	2	36	86	0	13	146	0	6	159	1	145	13	0
paad	145	59	5	1	9	47	1	32	4	1	33	4	1	25	17	0	11	39	0	7	62	2	56	4	2
prad	332	94	2	1	8	25	6	18	11	1	17	11	0	7	23	0	2	37	2	18	37	1	89	4	0
skcm	278	139	15	12	112	44	54	137	6	16	140	4	6	44	61	10	111	26	9	35	91	5	142	19	11
thca	397	7	3	1	1	3	1	11	0	0	11	0	1	7	1	0	3	67	1	15	5	0	7	4	1

### 3.4 Increased expression of FAM83 family genes is associated with poor survival

**Figure 4:**
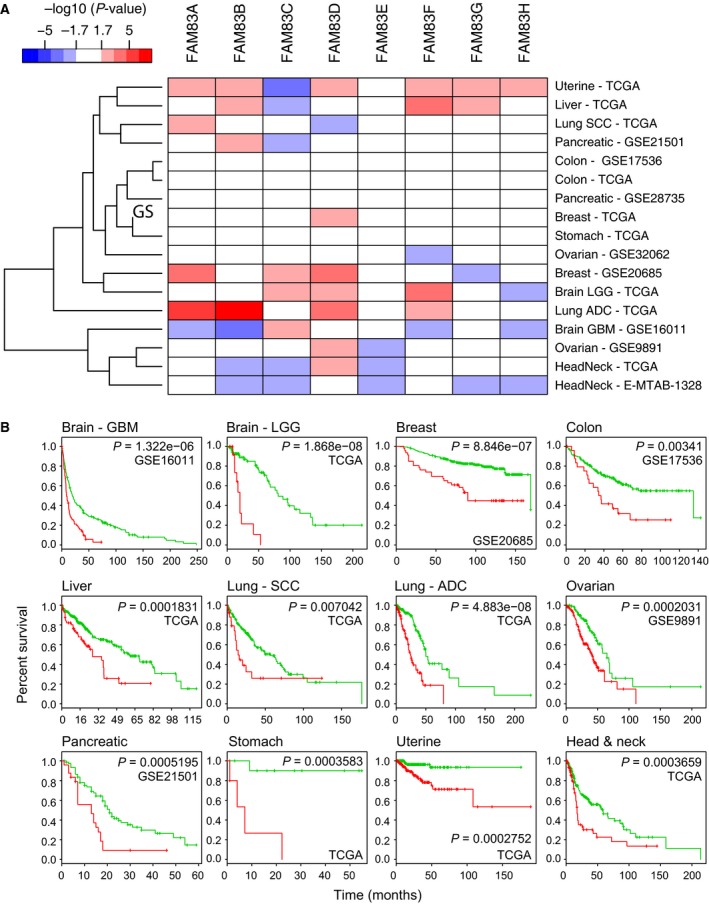
Increased expression of FAM83 family genes is associated with poor survival. (A) Heatmap of the *P*‐values for disease‐free survival for individual FAM83 family genes across 17 independent tumor data sets. Genes significantly associated with poor disease‐free survival when upregulated (*P *< 0.02) are shown in red, while genes significantly associated with poor disease‐free survival when downregulated (*P *< 0.02) are shown in blue. (B) For each tumor type, survival risk curves are shown; low and high risks are drawn in green and red, respectively. The *P*‐value represents the equality of survival curves based on a log‐rank test.

To investigate whether individual FAM83 family genes were associated with disease‐free survival, a log‐rank test was performed and the split‐point of the patient cohort was chosen where the *P*‐value between two patient groups was lowest. A heatmap of the *P*‐values of the prognostic property of each gene across 10 tumor types (17 total data sets) is shown in Fig. 4. In uterine cancer, increased expression levels of FAM83A, B, D, and F‐H were associated with decreased survival (Fig. 4A and Table S2; *P *< 0.02). Increased FAM83D gene expression was associated with decreased disease‐free survival in six tumor types (Fig. 4A and Table S2; *P *< 0.02). However, in general, individual FAM83 family genes, with the exception of FAM83D, were poor indicators of disease‐free survival. We then asked whether a FAM83 family gene expression signature served as a better marker for prognosis across 12 tumor types. Indeed, a significant association of the FAM83 signature with disease‐free survival was found for all 12 tumor types (0.007* *< *P*‐value* *< 1.868E‐08; Fig. 4B and Table S2). The signature was better able to predict survival compared to any individual FAM83 gene for all tumor types except lung cancer. These results indicate that overexpression of the FAM83 family signature is broadly predictive for disease‐free survival for the 12 tumor types.

### 3.5 Alterations of FAM83 family members are associated with key events in human breast cancers

**Figure 5:**
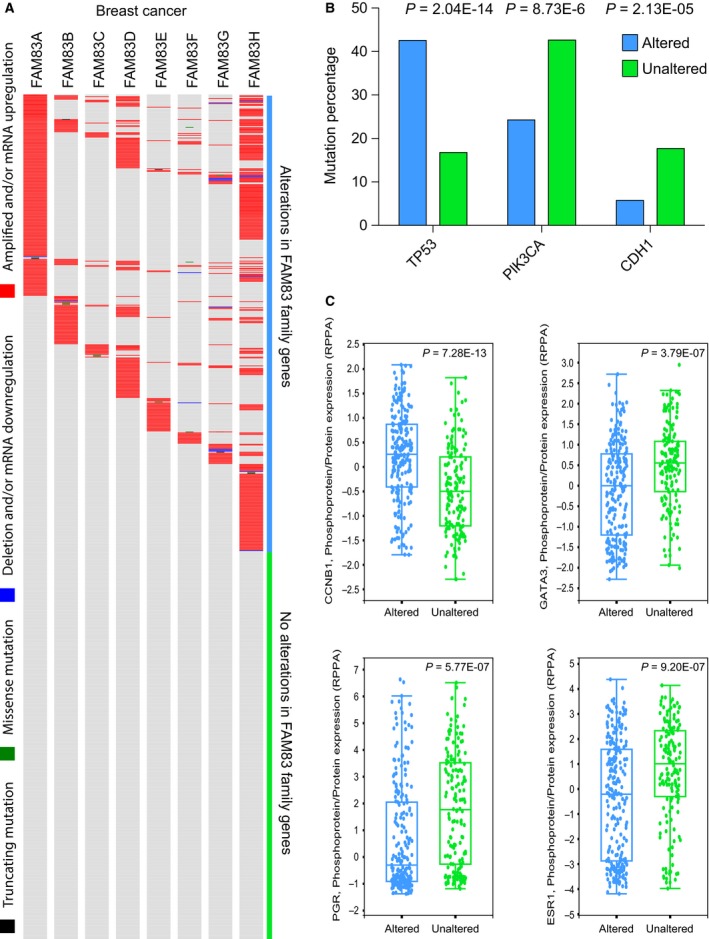
FAM83 family genes are frequently altered in breast cancer. (A) Spectrum of aberrations in FAM83 family genes in 960 human breast cancer samples. DNA copy number amplifications and/or gene expression upregulation are shown in red, DNA copy number loss and/or gene expression downregulation are shown in blue, and missense and truncating mutations are shown in green and black, respectively. (B) The percentage of breast cancer samples with TP53, PIK3CA, and CDH1 mutations in tumors with an alteration in at least one FAM83 family member (blue) and tumors without alteration in FAM83 genes (green). (C) Protein levels of CCNB1, GATA3, PGR, and ESR1 in breast cancer samples with an alteration in at least one FAM83 family member (blue) and samples without alteration in FAM83 genes (green).

As we observed a strong association between gene expression and DNA copy number in breast cancer across all FAM83 family members, we chose to further investigate the functional consequences of tumors with FAM83 family gene alterations in breast cancer. We investigated the types of aberrations in FAM83 family genes in 960 breast tumor samples (TCGA) including changes in gene expression, mutations, and DNA copy number changes. FAM83 family member gene expression was significantly different among breast cancer molecular subtypes (Fig. S3). Interestingly, more than half of all tumors harbored at least one alteration in a FAM83 family gene, with amplification and/or overexpression being the most common aberration (Fig. 5A). To search for the mechanisms for FAM83 family members contributing to tumor development, we investigated whether *mutations* in any genes were enriched in tumors with alterations of FAM83 family members and found that tumors with alterations in FAM83 family members were significantly more likely to also have a TP53 mutation (*P *= 2.04E‐14), whereas they were significantly less likely to have mutations in PIK3CA (*P *= 8.73E‐6) and CDH1 (E‐cadherin) (*P *= 2.13E‐05) (Fig. 5B; Table S3). Finally, we investigated whether protein expression of any genes was significantly different between the breast cancer group with FAM83 family alterations and those without any alteration. A significant difference was observed in expression of 55 proteins (Table S3; adjusted *P *< 0.05). For example, protein levels of CCNB1 (cyclin B1) were significantly higher (*P *= 7.28E‐13) in breast tumors with alterations in FAM83 family genes, whereas protein levels of GATA3 (*P *= 3.79E‐07), PGR (*P *= 5.77E‐07), and ESR1 (*P *= 9.20E‐07) were significantly lower (Fig. 5C). Gene ontology analysis of all 55 proteins revealed significant enrichment of apoptosis (*P *= 6.48E‐13), cell surface receptor signaling (*P *= 2.02E‐11), protein phosphorylation (*P *= 9.17E‐12) processes, and protein kinase activity (*P *= 1.95E‐08) (Table S4).

## 4 Discussion

FAM83 family oncogenes were recently discovered by two novel—yet very different—screens in the laboratories of Bissell and Jackson less than 5 years ago (Cipriano *et al*., 2012; Lee *et al*., 2012). These findings provided an important possible explanation for resistance of some patients to targeted tyrosine kinase inhibitor (TKI) drugs such as lapatinib (Lee *et al*., 2012). Since then, there have been only a couple of additional published studies (Cipriano *et al*., 2014a,b; Kuga *et al*., 2016; Liao *et al*., 2015; Mao *et al*., 2016; Okabe *et al*., 2015; Wang *et al*., 2013b, 2015). Here, we used a systematic genomics approach to assess oncogenic properties of the entire FAM83 family genes across human tumor types. FAM83A was found to be upregulated in lung, ovarian, pancreatic, and certain brain tumors (Fig. 1). A recent RNAseq analysis of lung adenocarcinomas also found FAM83A among genes with the largest fold‐change difference between tumor and paired normal samples (Li *et al*., 2015). In addition, overexpression of FAM83A increases cancer cell proliferation and invasion, phosphorylates c‐RAF and PI3K p85, upstream of MAPK and downstream of EGFR, and confers resistance to EGFR‐TKI (Lee *et al*., 2012; Li *et al*., 2015).

Our multi‐omics analysis showed that FAM83B was upregulated in lung squamous cell carcinoma (SCC), but not any other tumor type examined. Upregulation of FAM83B in lung SCC was observed also in a recent study that identified FAM83B as a novel biomarker for diagnosis and prognosis of lung SCC (Okabe *et al*., 2015). However, Cipriano *et al*. (2012) had shown that FAM83B gene expression was increased relative to relevant normal tissues in a number of cancers, including breast, lung, ovary, cervical, testis, thyroid, bladder, and lymphoid cancers (Cipriano *et al*., 2012). In the same study, it was shown that FAM83B could drive transformation of immortalized human mammary epithelial cells. In our analysis, comparing to normal mammary tissues, we did not detect any significant increase in expression of FAM83B in four independent breast tumor data sets; however, we did observe a significant association between FAM83B expression and its DNA copy number. One explanation for this discrepancy could be our relatively stringent criteria for increased expression in tumor versus normal (fold change 1.5 and adjusted *P*‐value < 0.05). Indeed, in one breast tumor data set (GSE10780), FAM83B is upregulated 1.4‐fold (adjusted *P*‐value* *= 0.001), just outside of our criteria for inclusion.

Our analysis also revealed that FAM83D was upregulated in the majority of tumor types examined in our study. We showed previously that higher FAM83D expression is significantly correlated with shorter survival in patients with breast (Walian *et al*., 2016). Furthermore, we showed that forced expression of FAM83D in MCF10A breast cells promoted cell proliferation, migration, and invasion, whereas FAM83D depletion by shRNA led to cell death at least in part through regulation of the tumor suppressor gene FBXW7 (Walian *et al*., 2016). Moreover, FAM83D expression is elevated in ovarian cancer (Ramakrishna *et al*., 2010), metastatic lung adenocarcinomas (Inamura *et al*., 2007), and hepatocellular carcinoma (Liao *et al*., 2015). In hepatocellular cell lines, FAM83D activates MEK/ERK signaling pathway (Wang *et al*., 2015).

FAM83H was found to be required for tooth enamel calcification (Lee *et al*., 2008), and mutations in FAM83H were shown to correlate with amelogenesis imperfecta (Zhang *et al*., 2015). An insertional mutagenesis screen to identify genes promoting prostate cancer in an orthotopic mouse model discovered FAM83H as a candidate oncogene (Nalla *et al*., 2016). Subsequent knockdown of FAM83H in LNCaP cells significantly inhibited colony formation (Nalla *et al*., 2016). A study in colorectal cancer identified FAM83H as an important regulator of keratin cytoskeletal organization, and that overexpression of FAM83H is accompanied by keratin filament disassembly and subsequently leads to loss of epithelial cell polarity (Kuga *et al*., 2013). In this analysis, we found that FAM83H was upregulated in the majority of tumor types. MYC, which is located directly upstream of FAM83H, is often amplified at the DNA copy number level in many tumor types. Given its proximity to MYC, increased copy number and expression of FAM83H could be a result of this aberration. However, we found no correlation between MYC and FAM83H gene expression in breast, ovarian, and prostate cancer (Pearson correlation < 0.25), suggesting strongly that FAM83H by itself is an important driver gene on chromosome 8q.

Not much is known about the role of FAM83 family members C, E, F, and G in cancer progression. FAM83C and E expression was increased in bladder and ovarian cancer and their overexpression was shown to promote human mammary epithelial cell transformation, respectively (Cipriano *et al*., 2014b). In a recent study, it was found that FAM83F expression was increased in esophageal SCC, and introduction of miR‐143 into esophageal cancer cells downregulated FAM83F expression, which results in inhibition of cell proliferation, migration, and invasion (Mao *et al*., 2016). Esophageal cancer was not included in our analysis. However, we found that FAM83F was upregulated in head‐and‐neck, breast, and lung cancer and that its expression was correlated with DNA copy number in eight of 19 tumor types. We also observed that increased expression of FAM83F was associated with poor patient survival in uterine, liver, low‐grade glioma, and lung adenocarcinoma. We could not find any literature with regard to the role of FAM83G in cancer progression; however, Vogt *et al*. (2014) showed that FAM83G is a substrate for type I bone morphogenetic protein (BMP) receptors and modulates BMP signaling (Vogt *et al*., 2014), which are known to play important roles in tumorigenesis and metastasis (Bailey *et al*., 2007; Yamamoto *et al*., 2002).

Despite the fact that the FAM83 family is a rather recent discovery of an oncogene family, the current literature suggests that they play a prominent role in many human cancer types. Our multi‐omics analyses lend further support for this. However, the family requires additional studies to clarify the mechanisms of action of individual FAM83 members. Moreover, Lee *et al*. (2012) has shown that TKIs amplified the expression of FAM83A (Lee *et al*., 2012), indicating that the activation of this oncogene family plays an important role in acquired drug resistance. In conclusion, our multi‐omics analysis of FAM83 family members opens up a new horizon for further understanding the significance of FAM83 genes in cancer and clinical applications in diagnosis, prognosis, and therapy.

## Author contributions

AMS, MJB, and JHM conceived and designed the project. AMS, SYL, BH, WH, and JHM analyzed and interpreted the data. AMS, MJB, and JHM wrote the manuscript. All authors read, edited, and approved the final manuscript.

## Supporting information


**Fig. S1.** Multi‐omic approach to investigate role of FAM83A‐H in cancer.

**Fig. S2.** Comparison of FAM83 family gene mRNA and protein levels in different normal human tissues.

**Fig. S3.** Comparison of FAM83 family gene expression across breast cancer molecular subtypes.

**Table S1.** FAM83 family gene Ttranscript level differences between tumor and normal tissues.

**Table S2.** *P*‐values of the prognostic property of each FAM83 gene and the FAM83 gene signature across 10 tumor types.

**Table S3.** Proteins differentially expressed between tumors with FAM83 mutation and those without any FAM83 alteration.

**Table S4.** Gene Ontology analysis of proteins differentially expressed between tumors with FAM83 mutation and those without any FAM83 alteration.

## References

[mol212016-bib-0001] Aguirre‐Gamboa R , Gomez‐Rueda H , Martinez‐Ledesma E , Martinez‐Torteya A , Chacolla‐Huaringa R , Rodriguez‐Barrientos A , Tamez‐Pena JG and Trevino V (2013) SurvExpress: an online biomarker validation tool and database for cancer gene expression data using survival analysis. PLoS One 8, e74250. 24066126 10.1371/journal.pone.0074250 PMC3774754

[mol212016-bib-0002] Bailey JM , Singh PK and Hollingsworth MA (2007) Cancer metastasis facilitated by developmental pathways: Sonic hedgehog, Notch, and bone morphogenic proteins. J Cell Biochem 102, 829–839. 17914743 10.1002/jcb.21509

[mol212016-bib-0003] Carithers LJ , Ardlie K , Barcus M , Branton PA , Britton A , Buia SA , Compton CC , DeLuca DS , Peter‐Demchok J , Gelfand ET *et al* (2015) A novel approach to high‐quality postmortem tissue procurement: the GTEx project. Biopreserv Biobank 13, 311–319. 26484571 10.1089/bio.2015.0032 PMC4675181

[mol212016-bib-0004] Cerami E , Gao J , Dogrusoz U , Gross BE , Sumer SO , Aksoy BA , Jacobsen A , Byrne CJ , Heuer ML , Larsson E *et al* (2012) The cBio cancer genomics portal: an open platform for exploring multidimensional cancer genomics data. Cancer Discov 2, 401–404. 22588877 10.1158/2159-8290.CD-12-0095 PMC3956037

[mol212016-bib-0005] Cipriano R , Bryson BL , Miskimen KL , Bartel CA , Hernandez‐Sanchez W , Bruntz RC , Scott SA , Lindsley CW , Brown HA and Jackson MW (2014a) Hyperactivation of EGFR and downstream effector phospholipase D1 by oncogenic FAM83B. Oncogene 33, 3298–3306. 23912460 10.1038/onc.2013.293 PMC3923847

[mol212016-bib-0006] Cipriano R , Graham J , Miskimen KL , Bryson BL , Bruntz RC , Scott SA , Brown HA , Stark GR and Jackson MW (2012) FAM83B mediates EGFR‐ and RAS‐driven oncogenic transformation. J Clin Investig 122, 3197–3210. 22886302 10.1172/JCI60517 PMC3428078

[mol212016-bib-0007] Cipriano R , Miskimen KL , Bryson BL , Foy CR , Bartel CA and Jackson MW (2014b) Conserved oncogenic behavior of the FAM83 family regulates MAPK signaling in human cancer. Mol Cancer Res 12, 1156–1165. 24736947 10.1158/1541-7786.MCR-13-0289 PMC4135001

[mol212016-bib-0008] Gao J , Aksoy BA , Dogrusoz U , Dresdner G , Gross B , Sumer SO , Sun Y , Jacobsen A , Sinha R , Larsson E *et al* (2013) Integrative analysis of complex cancer genomics and clinical profiles using the cBioPortal. Sci Signal 6, pl1. 23550210 10.1126/scisignal.2004088 PMC4160307

[mol212016-bib-0009] Inamura K , Shimoji T , Ninomiya H , Hiramatsu M , Okui M , Satoh Y , Okumura S , Nakagawa K , Noda T , Fukayama M *et al* (2007) A metastatic signature in entire lung adenocarcinomas irrespective of morphological heterogeneity. Hum Pathol 38, 702–709. 17376511 10.1016/j.humpath.2006.11.019

[mol212016-bib-0010] Kim MS , Pinto SM , Getnet D , Nirujogi RS , Manda SS , Chaerkady R , Madugundu AK , Kelkar DS , Isserlin R , Jain S *et al* (2014) A draft map of the human proteome. Nature 509, 575–581. 24870542 10.1038/nature13302 PMC4403737

[mol212016-bib-0011] Kuga T , Kume H , Kawasaki N , Sato M , Adachi J , Shiromizu T , Hoshino I , Nishimori T , Matsubara H and Tomonaga T (2013) A novel mechanism of keratin cytoskeleton organization through casein kinase Ialpha and FAM83H in colorectal cancer. J Cell Sci 126, 4721–4731. 23902688 10.1242/jcs.129684

[mol212016-bib-0012] Kuga T , Sasaki M , Mikami T , Miake Y , Adachi J , Shimizu M , Saito Y , Koura M , Takeda Y , Matsuda J *et al* (2016) FAM83H and casein kinase I regulate the organization of the keratin cytoskeleton and formation of desmosomes. Sci Rep 6, 26557. 27222304 10.1038/srep26557 PMC4879633

[mol212016-bib-0013] Lee SK , Hu JC , Bartlett JD , Lee KE , Lin BP , Simmer JP and Kim JW (2008) Mutational spectrum of FAM83H: the C‐terminal portion is required for tooth enamel calcification. Hum Mutat 29, E95–E99. 18484629 10.1002/humu.20789 PMC2889227

[mol212016-bib-0014] Lee SY , Meier R , Furuta S , Lenburg ME , Kenny PA , Xu R and Bissell MJ (2012) FAM83A confers EGFR‐TKI resistance in breast cancer cells and in mice. J Clin Investig 122, 3211–3220. 22886303 10.1172/JCI60498 PMC3428077

[mol212016-bib-0015] Li Y , Xiao X , Ji X , Liu B and Amos CI (2015) RNA‐seq analysis of lung adenocarcinomas reveals different gene expression profiles between smoking and nonsmoking patients. Tumour Biol 36, 8993–9003. 26081616 10.1007/s13277-015-3576-y PMC4674426

[mol212016-bib-0016] Liao W , Liu W , Liu X , Yuan Q , Ou Y , Qi Y , Huang W , Wang Y and Huang J (2015) Upregulation of FAM83D affects the proliferation and invasion of hepatocellular carcinoma. Oncotarget 6, 24132–24147. 26125229 10.18632/oncotarget.4432 PMC4695175

[mol212016-bib-0017] Mao Y , Liu J , Zhang D and Li B (2016) miR‐143 inhibits tumor progression by targeting FAM83F in esophageal squamous cell carcinoma. Tumour Biol 37, 9009–9022. 26758433 10.1007/s13277-015-4760-9

[mol212016-bib-0018] Meinkoth J , Killary AM , Fournier RE and Wahl GM (1987) Unstable and stable CAD gene amplification: importance of flanking sequences and nuclear environment in gene amplification. Mol Cell Biol 7, 1415–1424. 3600632 10.1128/mcb.7.4.1415-1424.1987 PMC365229

[mol212016-bib-0019] Mele M , Ferreira PG , Reverter F , DeLuca DS , Monlong J , Sammeth M , Young TR , Goldmann JM , Pervouchine DD , Sullivan TJ *et al* (2015) Human genomics. The human transcriptome across tissues and individuals. Science 348, 660–665. 25954002 10.1126/science.aaa0355 PMC4547472

[mol212016-bib-0020] Nalla AK , Williams TF , Collins CP , Rae DT and Trobridge GD (2016) Lentiviral vector‐mediated insertional mutagenesis screen identifies genes that influence androgen independent prostate cancer progression and predict clinical outcome. Mol Carcinog 55, 1761–1771. 26512949 10.1002/mc.22425 PMC5393267

[mol212016-bib-0021] Okabe N , Ezaki J , Yamaura T , Muto S , Osugi J , Tamura H , Imai J , Ito E , Yanagisawa Y , Honma R *et al* (2015) FAM83B is a novel biomarker for diagnosis and prognosis of lung squamous cell carcinoma. Int J Oncol 46, 999–1006. 25586059 10.3892/ijo.2015.2817 PMC4324586

[mol212016-bib-0022] Ramakrishna M , Williams LH , Boyle SE , Bearfoot JL , Sridhar A , Speed TP , Gorringe KL and Campbell IG (2010) Identification of candidate growth promoting genes in ovarian cancer through integrated copy number and expression analysis. PLoS One 5, e9983. 20386695 10.1371/journal.pone.0009983 PMC2851616

[mol212016-bib-0023] Snijders AM , Hermsen MA , Baughman J , Buffart TE , Huey B , Gajduskova P , Roydasgupta R , Tokuyasu T , Meijer GA , Fridlyand J *et al* (2008) Acquired genomic aberrations associated with methotrexate resistance vary with background genomic instability. Genes Chromosomes Cancer 47, 71–83. 17943968 10.1002/gcc.20509

[mol212016-bib-0024] Uhlen M , Fagerberg L , Hallstrom BM , Lindskog C , Oksvold P , Mardinoglu A , Sivertsson A , Kampf C , Sjostedt E , Asplund A *et al* (2015) Proteomics. Tissue‐based map of the human proteome. Science 347, 1260419. 25613900 10.1126/science.1260419

[mol212016-bib-0025] Vogt J , Dingwell KS , Herhaus L , Gourlay R , Macartney T , Campbell D , Smith JC and Sapkota GP (2014) Protein associated with SMAD1 (PAWS1/FAM83G) is a substrate for type I bone morphogenetic protein receptors and modulates bone morphogenetic protein signalling. Open Biol 4, 130210. 24554596 10.1098/rsob.130210 PMC3938053

[mol212016-bib-0026] Walian PJ , Hang B and Mao JH (2016) Prognostic significance of FAM83D gene expression across human cancer types. Oncotarget 7, 3332–3340. 26678035 10.18632/oncotarget.6620 PMC4823109

[mol212016-bib-0027] Wang J , Duncan D , Shi Z and Zhang B (2013a) WEB‐based GEne SeT AnaLysis Toolkit (WebGestalt): update 2013. Nucleic Acids Res 41, W77–W83. 23703215 10.1093/nar/gkt439 PMC3692109

[mol212016-bib-0028] Wang D , Han S , Peng R , Wang X , Yang XX , Yang RJ , Jiao CY , Ding D , Ji GW and Li XC (2015) FAM83D activates the MEK/ERK signaling pathway and promotes cell proliferation in hepatocellular carcinoma. Biochem Biophys Res Commun 458, 313–320. 25646692 10.1016/j.bbrc.2015.01.108

[mol212016-bib-0029] Wang Z , Liu Y , Zhang P , Zhang W , Wang W , Curr K , Wei G and Mao JH (2013b) FAM83D promotes cell proliferation and motility by downregulating tumor suppressor gene FBXW7. Oncotarget 4, 2476–2486. 24344117 10.18632/oncotarget.1581 PMC3926842

[mol212016-bib-0030] Yamamoto T , Saatcioglu F and Matsuda T (2002) Cross‐talk between bone morphogenic proteins and estrogen receptor signaling. Endocrinology 143, 2635–2642. 12072396 10.1210/endo.143.7.8877

[mol212016-bib-0031] Zhang B , Kirov S and Snoddy J (2005) WebGestalt: an integrated system for exploring gene sets in various biological contexts. Nucleic Acids Res 33, W741–W748. 15980575 10.1093/nar/gki475 PMC1160236

[mol212016-bib-0032] Zhang C , Song Y and Bian Z (2015) Ultrastructural analysis of the teeth affected by amelogenesis imperfecta resulting from FAM83H mutations and review of the literature. Oral Surg Oral Med Oral Pathol Oral Radiol 119, e69–e76. 25487982 10.1016/j.oooo.2014.09.002

