# Supplementary figures and images for: FAM83 family oncogenes are broadly involved in human cancers: an integrative multi‐omics approach
Source: Mol Oncol. 2017 Jan 9;11(2):167–79. doi: 10.1002/1878-0261.12016 (PMC5527452; doi:10.1002/1878-0261.12016)

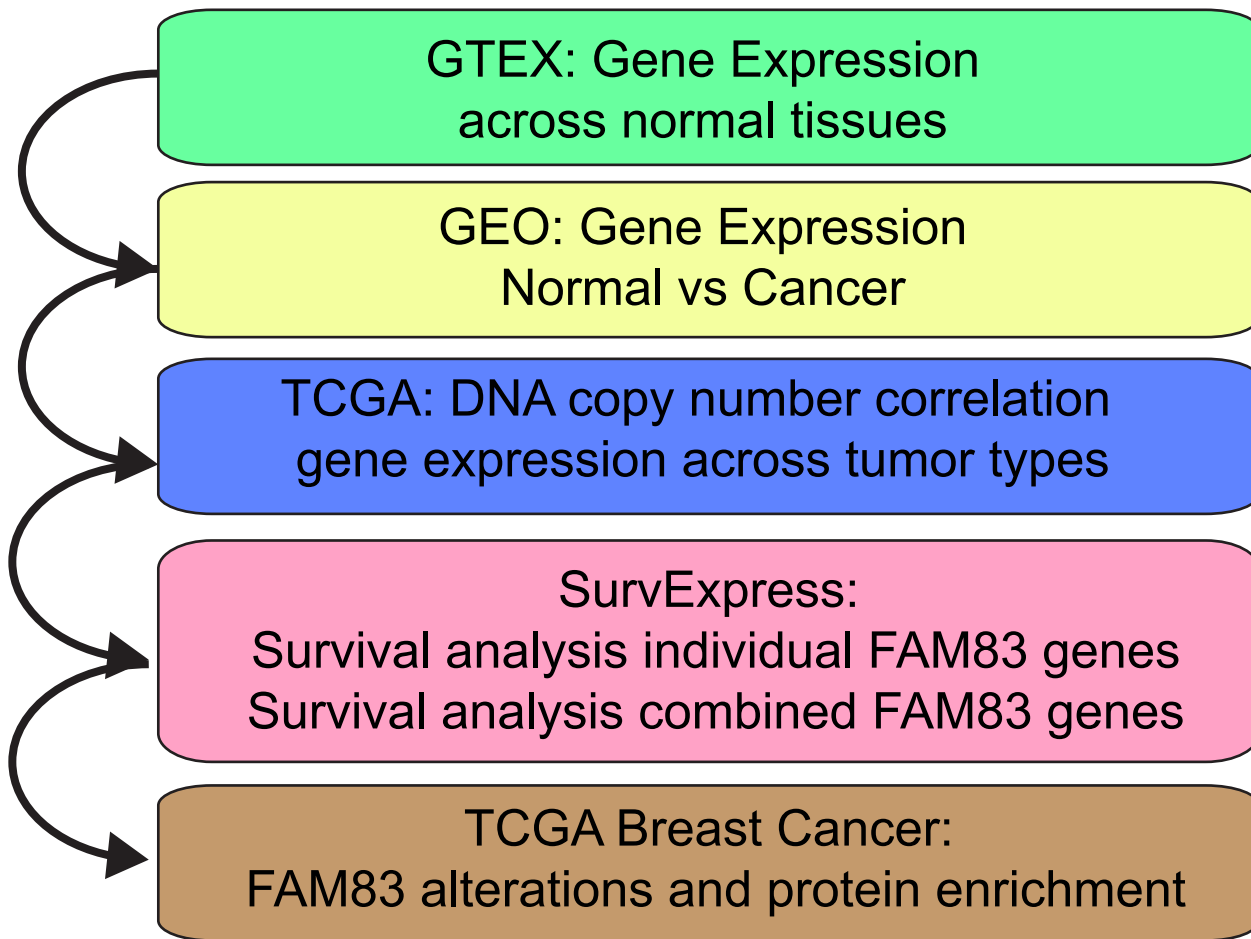

Figure S1. Multi-omic approach to investigate role of FAM83A-H in cancer

Supplement: Supplementary file 1 — Fig. S1. Multi‐omic approach to investigate role of FAM83A‐H in cancer. [file MOL2-11-167-s001.pdf]
