# Supplementary material for: FAM83 family oncogenes are broadly involved in human cancers: an integrative multi‐omics approach
Source: Mol Oncol. 2017 Jan 9;11(2):167–79. doi: 10.1002/1878-0261.12016 (PMC5527452; doi:10.1002/1878-0261.12016)
Supplement: Supplementary file 2 — Fig. S2. Comparison of FAM83 family gene mRNA and protein levels in different normal human tissues. [file MOL2-11-167-s002.pdf]

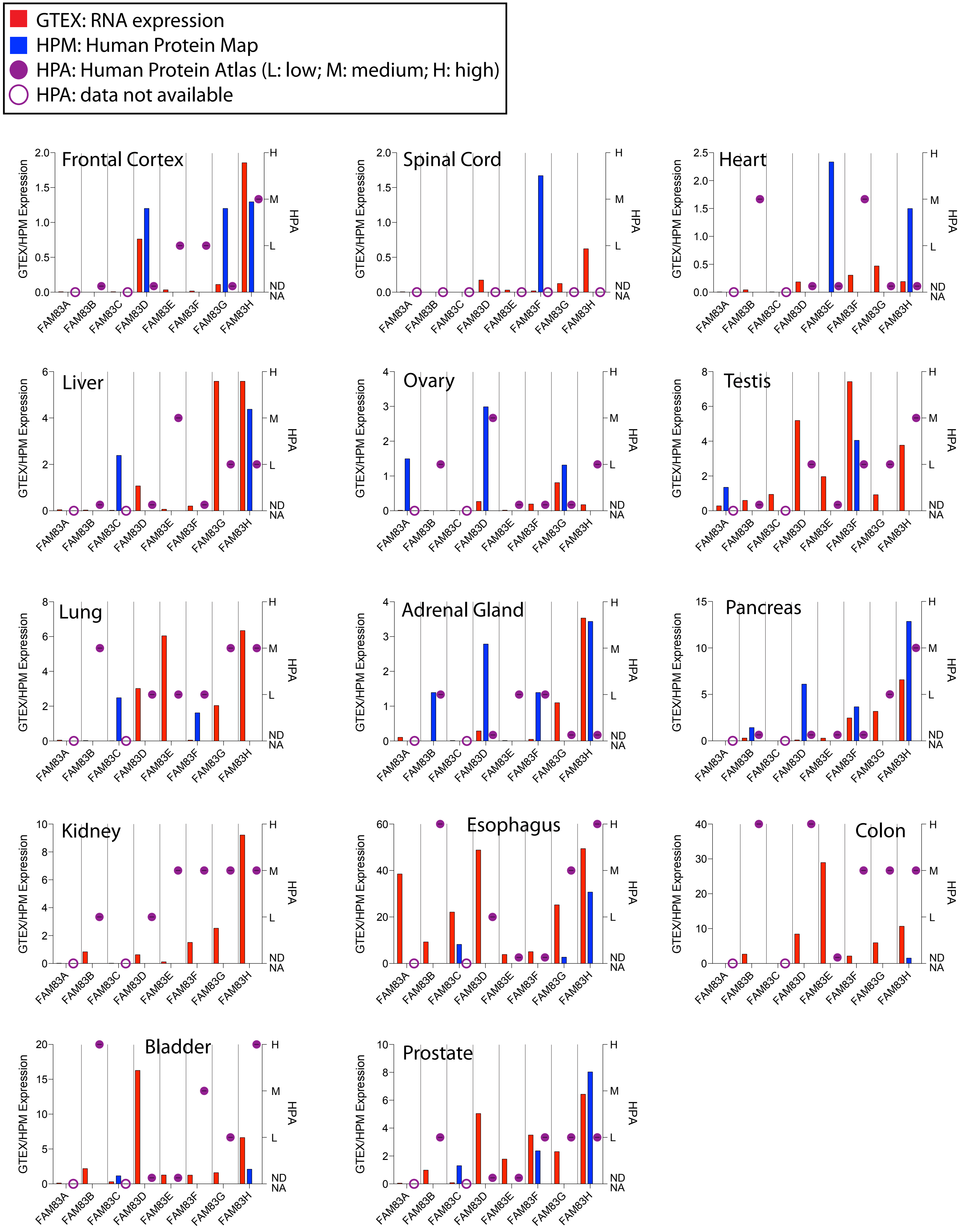

Figure S2. Comparison of FAM83 family gene mRNA and protein levels in different normal human tissues.
