# Supplementary material for: FAM83 family oncogenes are broadly involved in human cancers: an integrative multi‐omics approach
Source: Mol Oncol. 2017 Jan 9;11(2):167–79. doi: 10.1002/1878-0261.12016 (PMC5527452; doi:10.1002/1878-0261.12016)
Supplement: Supplementary file 3 — Fig. S3. Comparison of FAM83 family gene expression across breast cancer molecular subtypes. [file MOL2-11-167-s003.pdf]

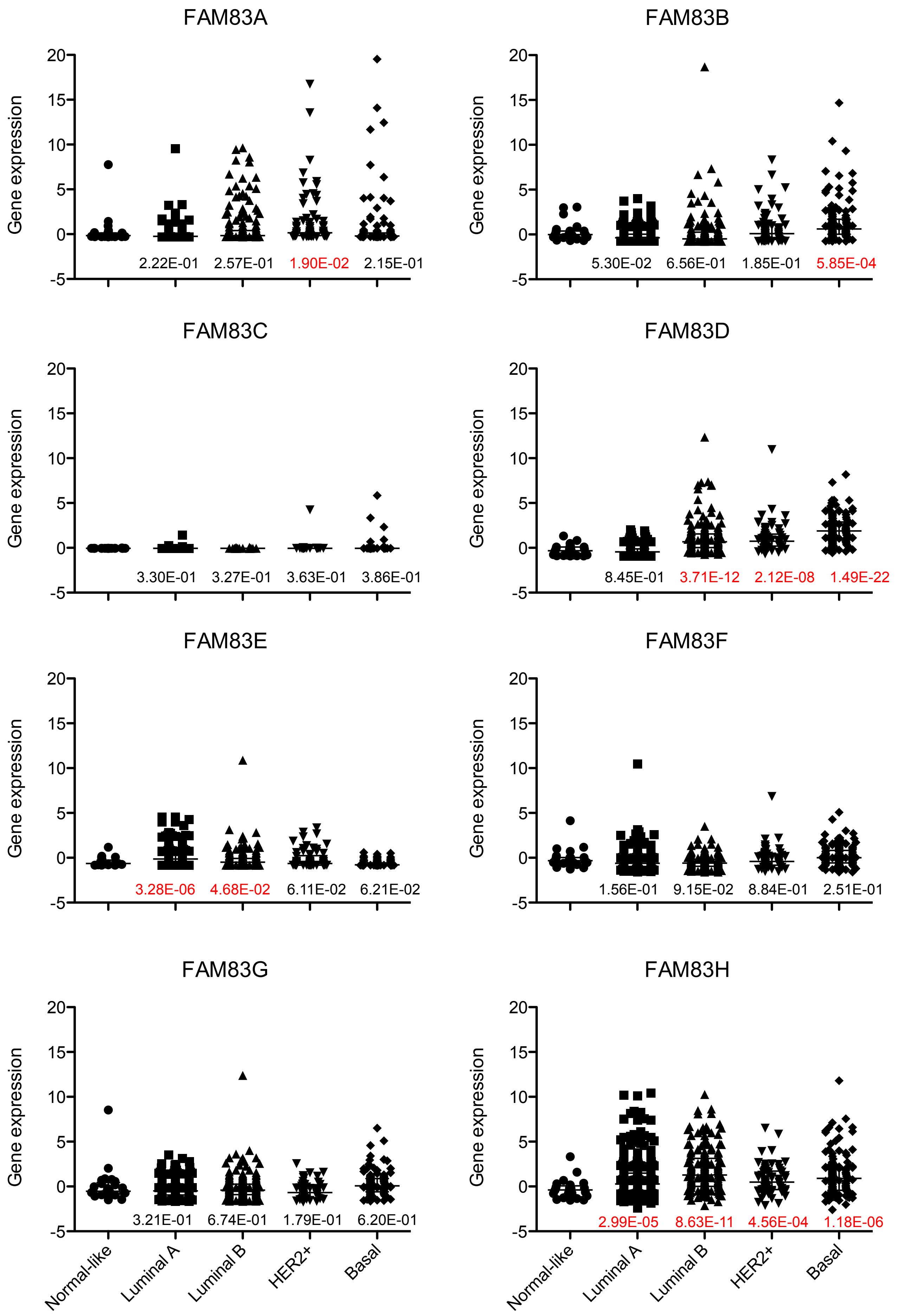

Figure S3. Comparison of FAM83 family gene expression across breast cancer molecular subtypes. The p-values represent significance of expression level difference between breast tumor subtypes and normal-like tumors.
